# Cytoplasmic amino acid profiles of clinical and ATCC 29213 strains of *Staphylococcus aureus* harvested at different growth phases

**DOI:** 10.17305/bb.2023.9246

**Published:** 2023-12-01

**Authors:** Mousa Alreshidi, Hugh Dunstan, Tim Roberts, Fayez Alreshidi, Ashfaque Hossain, Fevzi Bardakci, Mejdi Snoussi, Riadh Badraoui, Mohd Adnan, Sultan Alouffi, Mohd Saeed

**Affiliations:** 1 Department of Biology, College of Science, University of Ha’il, Ha’il, Saudi Arabia; 2 InnovAAte Pty Ltd, Newcastle, NSW, Australia; 3 Metabolic Research Group, Faculty of Science, School of Environmental and Life Sciences, University Drive, Callaghan, NSW, Australia; 4 Department of Family and Community Medicine, College of Medicine, University of Ha’il, Ha’il, Saudi Arabia; 5 Department of Medical Microbiology and Immunology, RAK Medical and Health Sciences University, RAK Hospital, Al Qusaidat, Ras Al Khaimah, United Arab Emirates; 6 Laboratory of Genetics, Biodiversity and Valorization of Bio-Resources (LR11ES41), Higher Institute of Biotechnology of Monastir, University of Monastir, Monastir, Tunisia; 7 Department of Clinical Laboratory Sciences, College of Applied Medical Sciences, University of Ha’il, Ha’il, Saudi Arabia

**Keywords:** Metabolic homeostasis, growth phases, bacterial adaptation, bacterial strains

## Abstract

*Staphylococcus aureus* strains are a great contributor to both hospital acquired infections as well as community acquired infections. The objective of the present investigation was to compare potential differences in cytoplasmic amino acid levels between clinical and ATCC 29213 strains of *S. aureus*. The two strains were grown under ideal conditions to mid-exponential and stationary growth phases, after which they were harvested to analyze their amino acid profiles. Initially, the amino acid patterns of both strains were compared at the mid-exponential phase when grown in controlled conditions. At the mid-exponential phase, both strains shared common features in cytoplasmic amino acid levels, with glutamic acid, aspartic acid, proline, and alanine identified as key amino acids. However, the concentration profiles of seven amino acids exhibited major variances between the strains, even though the total cytoplasmic levels of amino acids did not alter significantly. At the stationary phase, the magnitudes of the amino acids abundant in the mid-exponential phase were altered. Aspartic acid became the most abundant amino acid in both strains accounting for 44% and 59% of the total amino acids in the clinical and ATCC 29213 strains, respectively. Lysine was the second most abundant amino acid in both strains, accounting for 16% of the total cytoplasmic amino acids, followed by glutamic acid, the concentration of which was significantly higher in the clinical strain than in the ATCC 29213 strain. Interestingly, histidine was clearly present in the clinical strain but was virtually lacking in the ATCC 29213 strain. This study reveals the dynamic diversity of amino acid levels among strains, which is an essential step toward illustrating the variability in *S. aureus* cytoplasmic amino acid profiles and could be significant in explaining variances among strains of *S. aureus*.

## Introduction

*Staphylococcus aureus* has appeared as a main source of hospital and community-associated infections. The Centers for Disease Control and Prevention of the United States (US) have determined that *S. aureus* strains, in particular methicillin-resistant *S. aureus* strains (MRSA), are among the most dangerous pathogens causing high rates of illness and death globally. Several studies have been conducted with the intent of determining similarities and differences among different strains of *S. aureus* so that a novel antibiotic can be made. Metabolic changes play an important role in the effectiveness and efficacy of survival mechanisms used by different strains in response to modifications in environmental settings or oxidative stress [1, 2]. Recent studies indicate that the survival and persistence of *S. aureus* depend on its ability to induce the optimal metabolic homeostasis required for its growth in adjusted environmental conditions [1, 3–5]. A recent study showed that *S. aureus* adjusted its amino acid release and uptake through the different phases of replication [6]. The researchers interpreted this result as a survival strategy used by *S. aureus* strains to combat alterations in pH, osmotic pressure, and temperature. Amino acids play an important role in many aspects, including biofilm formation. It was demonstrated that the consumption of amino acids has varied between *S. aureus* grown in a biofilm in comparison to those grown in planktonic cultures [7]. A set of metabolic alterations observed in methicillin-susceptible *S. aureus* (MSSA) and MRSA following the exposure to a combination of antibiotics suggested a possible stress response mechanism in these strains’ metabolism [8]. Proteomic and genomic approaches determined significant variations of *S. aureus* strains [9–14]. *S. aureus* RN6390 and UAMS1 showed different proteomic profiles in response to oxidative stress [15]. A metabolomics approach showed that *S. aureus* COL and HG001 consumed and secreted different quantities of metabolites [16]. Another study compared the ability of a clinical isolate with a food isolate of *S. aureus* to form a biofilm, and the results showed that these strains produced different quantities of biofilm in response to chemical and physical stresses [17]. Previous studies of the clinical strain of *S. aureus*, studied in the present investigation, exhibited metabolic homeostasis responses to a range of various alterations in environmental conditions, ranging from extreme cold to more subtle variations similar to human skin and wound site [18, 19]. The results have shown that the clinical strain was able to rapidly adapt to alterations in environmental conditions by changing the cytoplasmic composition of metabolites and proteins. Therefore, this study was undertaken to determine whether the cytoplasmic amino acid responses differed between a clinical strain and the ATCC 29213 strain of *S. aureus*, following growth to mid-exponential and stationary phases. It was postulated that the clinical strain and the ATCC 29213 strain would have different amino acid responses when harvested at mid-exponential and stationary phases. These findings would contribute to finding similar and divergent amino acid perturbations of *S. aureus* strains, which may lead to the identification of strain-specific approaches to the establishment of novel antibacterial mechanisms.

## Materials and methods

### *S. aureus* strains

Two strains of *S. aureus,* including a clinical strain collected from a hospital [20] and a type strain *S. aureus* subspecies *aureus* Rosenbach (ATCC^®^ 29213), were observed. Both strains were regularly subjected to biochemical identification, using the API Staph (bioMerieux, Australia) and also molecular identification by amplifying 16S rRNA gene primers [21].

### Growth conditions

Both strains were incubated in overnight cultures under ideal conditions at 37 ^∘^C and continuous shaking at 120 rpm to be used the next day for conducting the experiments. Five mL of the overnight culture of each strain was transferred to conical flasks (500 mL) comprising 95 mL tryptic soy broth (TSB) culture media (Oxoid Ltd., Australia) and grown to mid-exponential or stationary phase prior harvesting for amino acid profiling. Bacterial replication was aseptically assessed by measuring the optical density at 600 nm to calculate cell numbers so that the strain cultures could be collected at either mid-exponential or stationary phase. The experiments were repeated several times to account for time-based differences, with a total of 12 and 6 replicates conducted during the mid-exponential phase for the clinical and ATCC 219213 strains, respectively. Bacterial cells were then separated from the media by centrifugation at 6500 × *g* for 25 min. The separated cells were then rinsed several times using phosphate-buffered saline (PBS) at 4 ^∘^C to remove the culture media from the bacterial cells. The rinsed cells were directly placed in liquid nitrogen to stop all metabolic processes followed by lyophilization process prior to being subjected to the extraction method for cytoplasmic amino acids analysis.

### Amino acids analysis

Metabolites in the cytoplasm were extracted from cells of both strains using the cold methanol/water method [22]. Lyophilized cells (≈10–12 mg) were mixed with extraction buffer (1:1 ice cold methanol/water), placed in liquid nitrogen and kept at −20 ^∘^C for 30 min to allow the solution to gradually thaw. The mixtures were then transferred to Falcon tubes and placed in the centrifuge to separate the extract from the cell debris. The supernatant containing the metabolites was placed into new tubes and dried in a centrifugal vacuum dryer (Labconco CentriVap) to remove the methanol/water. The cytoplasmic amino acids were extracted and processed using the Phenomenex EZ:faast analytical kit as per manufacturer’s instructions for analysis by gas chromatography flame ionization (GC-FID) (Agilent, Hewlett Packard HP 6890 series) as formerly described [5].

### Statistical analysis

The acquired amino acid data gained with the GC-FID instrument were transferred to a Microsoft Excel spreadsheet. The transferred data were subsequently submitted to the MetaboAnalyst version 5.0 web-based platform (www.metaboanalyst.ca) [23] to process the cytoplasmic amino acid data and create figures. Prior to the analysis, the complete dataset was subjected to normalization processes to achieve better analysis. A heatmap was generated using a Euclidean distance measurement with a Ward clustering algorithm. The volcano analysis was conducted to determine the significant amino acids between the two strains based on the fold change (≥2) and *t*-tests. Multivariate analysis, including the unsupervised method of principal component analysis (PCA) and the supervised model of partial least squares-discriminant analysis (PLS-DA), was performed to determine and visualize the differences in amino acid patterns between the clinical and ATCC 29213 strains. PCA presents a data overview and the best direction for explaining the variances in the dataset, whereas PLS-DA identifies the most influential amino acids using variable importance projection (VIP). Amino acids with a VIP score greater than 1 were considered significantly important amino acids for distinguishing between the examined strains.

**Figure 1.**
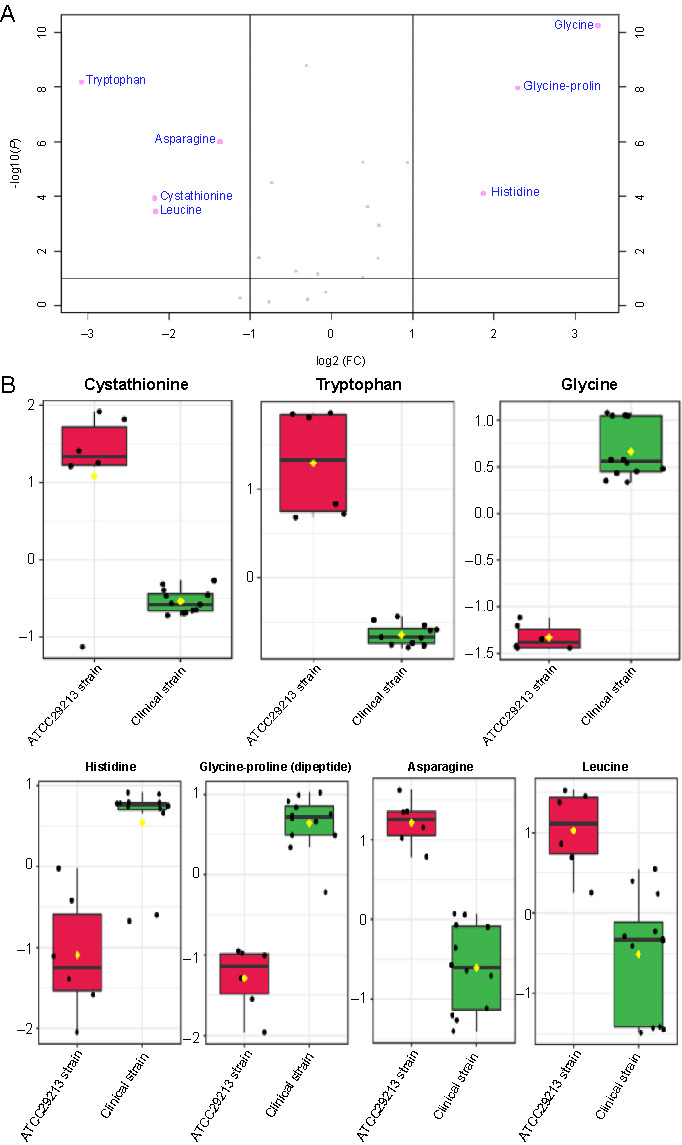
(A) Volcano plot showing the distribution of amino acid concentrations, fold changes, and *P* values. Significant amino acids with fold change > |2| and *P* value < 0.01 are labeled, namely, glycine, proline–glycine, histidine, tryptophan, asparagine, cystathionine, and leucine; (B) Boxplots show each significantly altered amino acid *(P* < 0.01) extracted from the clinical strain (green) and the ATCC29213 strain (red) grown to exponential phase, which were all derived from the volcano plot. The *x*-axis shows the specific amino acid, and the *y*-axis is the normalized concentration. FC: Fold change.

## Results

### Mid-exponential phase analysis

The clinical and ATCC 29213 strains of *S. aureus* were cultured in TSB to mid-exponential phase under controlled parameters to investigate the differences between these strains at the amino acids level. The analyses found the existence of 22 amino acids and amino acid derivatives that could be detected by the GC-FID method. The most abundant cytoplasmic amino acid in both strains was glutamic acid, but the ATCC 29213 strain had 45% more of this amino acid in the cytoplasm than the clinical strain. This accounted for most of the measured increase in the total cytoplasmic amino acids in the ATCC 29213 strain, even though this difference in total amino acids was not statistically significant. Aspartic acid was the next most abundant amino acid in the cytoplasm; its level was almost identical in both strains under these optimal conditions of the mid-exponential phase. A volcano plot was used to visualize the statistical results of amino acid levels among the tested *S. aureus* strains (Figure 1A). Amino acids with twofold changes on the *x*-axis and *P* < 0.01 on the *y*-axis were measured as significantly altered. The amino acids that had substantially increased and decreased in the clinical strain compared to the ATCC 29213 strain were displayed on the right and left sides of the volcano plot, respectively, while amino acids that did not show significant changes appeared in the center of the plot (in gray). Subsequently, the box and whisker plots were generated for the amino acids that differed between the two strains (Figure 1B). Glycine, histidine, and glycine–proline (dipeptide) were significantly elevated in the clinical strain compared with the ATCC 29213 strain, whereas tryptophan, leucine, cystathionine, and asparagine were significantly decreased, while 15 amino acids demonstrated no statistical changes between the strains (Figure 1B).

**Figure 2.**
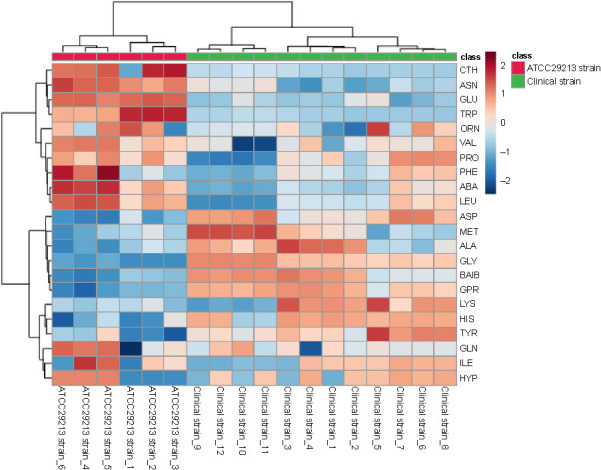
**Heatmap of cytoplasmic amino acid data of cells collected at mid-exponential phase.** Rows and columns denote amino acids and the replicates for each strain, respectively. The color inside the heatmap represents the relative fold change of each amino acid between strains: Red and blue colors represent higher and lower abundance, respectively. CTH: Cystathionine; ASN: Asparagine; GLU: Glutamic acid; TRP: Tryptophan; ORN: Ornithine; VAL: Valine; PRO: Proline; PHE: Phenylalanine; ABA: α-aminobutyric acid; LEU: Leucine; ASP: Aspartic acid: MET: Methionine; ALA: Alanine; GLY: Glycine; BAIB: β-aminoisobutyric acid; LYS: Lysine; HIS: Histidine; TYR: Tyrosine; GLN: Glutamine; ILE: Isoleucine; HYP: Hydroxiproline; GPR: Glycine-proline (dypeptide).

Heatmaps and hierarchical clustering of the amino acid data provided a visualization of the amino acids abundance in each sample of the clinical and ATCC 29213 strains, showing the specific concentrations of each amino acid in all samples (Figure 2). The color of the heatmap represents the relative fold change of each amino acid between the clinical strain and the ATCC 29213 strain, with red and blue colors indicating higher and lower levels, respectively. The heatmap clustering was calculated using two methods, the Euclidean distance and the Ward clustering method. This statistical technique clearly showed that the examined strains could be separated into two groups based on their amino acid profiles. Heatmap analysis demonstrated a general reduction in cystathionine, asparagine, and tryptophan in all clinical strain replicates in comparison to the ATCC 29213 strain replicates. However, an increase in glycine and dipeptide glycine–proline was noted in all clinical strain replicates in comparison to the ATCC 29213 strain.

**Figure 3.**
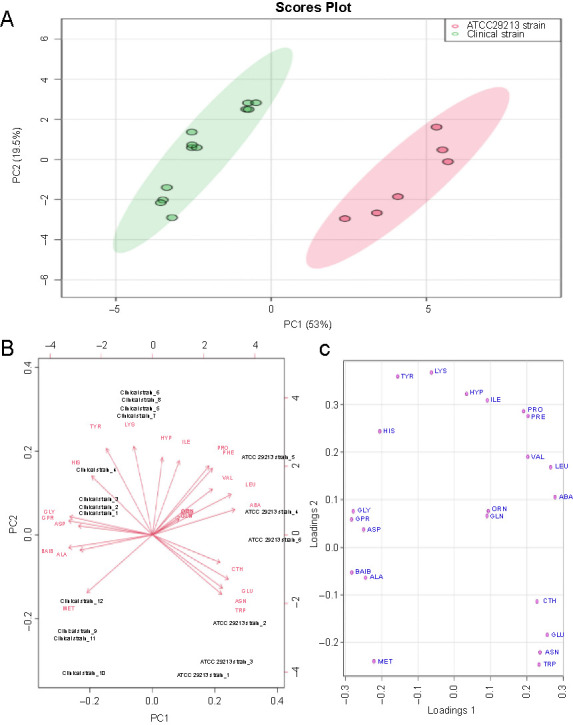
(A) PCA scores (PC1 vs PC2) plotted from cytoplasmic amino acid data analyzed from the mid-exponential phase for clinical (green oval) and ATCC 29213 (pink oval) strains of *S. aureus*. The *S. aureus* strains were cultured in liquid media to mid-exponential phase of growth prior extraction of amino acid metabolites and analyses by GC-FID; (B and C) Loadings plot and biplot derived from the PCA model. PCA: Principal component analysis; GC-FID: Gas chromatography flame ionization; CTH: Cystathionine; ASN: Asparagine; GLU: Glutamic acid; TRP: Tryptophan; ORN: Ornithine; VAL: Valine; PRO: Proline; PHE: Phenylalanine; ABA: α-aminobutyric acid; LEU: Leucine; ASP: Aspartic acid: MET: Methionine; ALA: Alanine; GLY: Glycine; BAIB: β-aminoisobutyric acid; LYS: Lysine; HIS: Histidine; TYR: Tyrosine; GLN: Glutamine; ILE: Isoleucine; HYP: Hydroxiproline; GPR: Glycine-proline (dypeptide).

**Figure 4.**
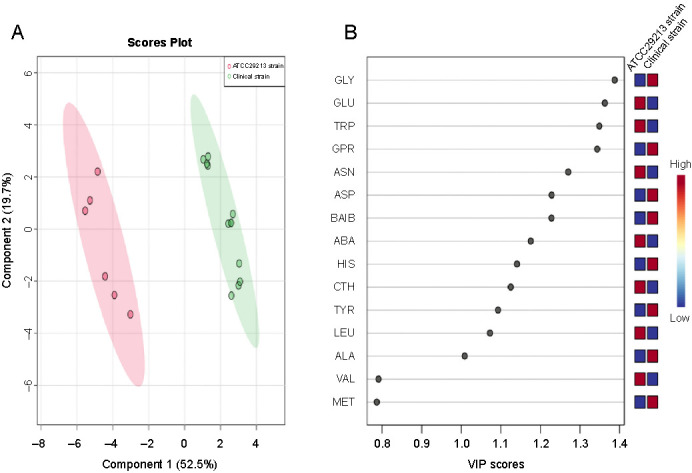
(A) PLS-DA of *S. aureus* amino acid profiles derived from the clinical and ATCC 29213 strains harvested at the mid-exponential phase. Each sample replicate is represented on the plot as a dot, clinical strain replicates are colored green and ATCC 29213 strain replicates are colored pink; (B) VIP scores show the most important amino acids contributing to the distinguishment of the two strains. A VIP score > 1 indicates that those amino acids greatly affected the PLS-DA model. The relative abundance of amino acids is presented by a colored scale from blue to red representing low to high abundance, respectively. PLS-DA: Partial least squares-discriminant analysis; VIP: Variable importance projection; CTH: Cystathionine; ASN: Asparagine; GLU: Glutamic acid; TRP: Tryptophan; VAL: Valine; ABA: α-aminobutyric acid; LEU: Leucine; ASP: Aspartic acid; MET: Methionine; ALA: Alanine; GLY: Glycine; BAIB: β-aminoisobutyric acid; HIS: Histidine; TYR: Tyrosine; GPR: Glycine-proline (dypeptide).

Pattern recognition techniques, including unsupervised PCA and supervised PLS-DA, were undertaken to further investigate whether differences in the cytoplasmic amino acid profiles exist among *S. aureus* strains harvested at the mid-exponential phase. The PCA and PLS-DA models were constructed after normalization was performed by sum, followed by log transformation and auto-scaling (mean-centered and divided by the standard deviation of each variable). The quality parameters of the PLS-DA model were as follows: R2 ═ 0.942 and Q2 ═ 0.906. Both two-dimensional plots of the PCA and PLS-DA models confirmed that the amino acid profiles of the clinical strain were clearly different from those of the ATTC 29213 strain. The amino acid profiles obtained from the clinical strain were positioned in the negative PC1 direction and those obtained from the ATCC 29213 strain were placed in the positive PC1 direction, as shown in the biplot and PCA plots (Figure 3A and 3B). The scores of the PCA and PLS-DA plots explained >70% of the total variance (Figures 3 and 4). The results indicate that the multivariate pattern recognition methods created a noticeable representation of the amino acid profiles associated with the *S. aureus* strains. The samples from each strain were tightly clustered and well separated from each other based on their amino acid profiles, representing clear differences in homeostasis between the strains. The loadings plot and biplot indicated the contribution of each amino acid to the PCA model. The VIP plot (Figure 4), derived from PLS-DA, represented the essential amino acids in the PLS-DA model. A VIP score > 1 indicates that those amino acids greatly affect the PLS-DA model. The colored boxes on the right indicate the concentrations of the corresponding amino acid in each strain. PLS-DA indicated that 13 amino acids in both strains had VIP scores > 1.0. The experiments were repeated several times for both strains to ensure reproducibility.

**Figure 5.**
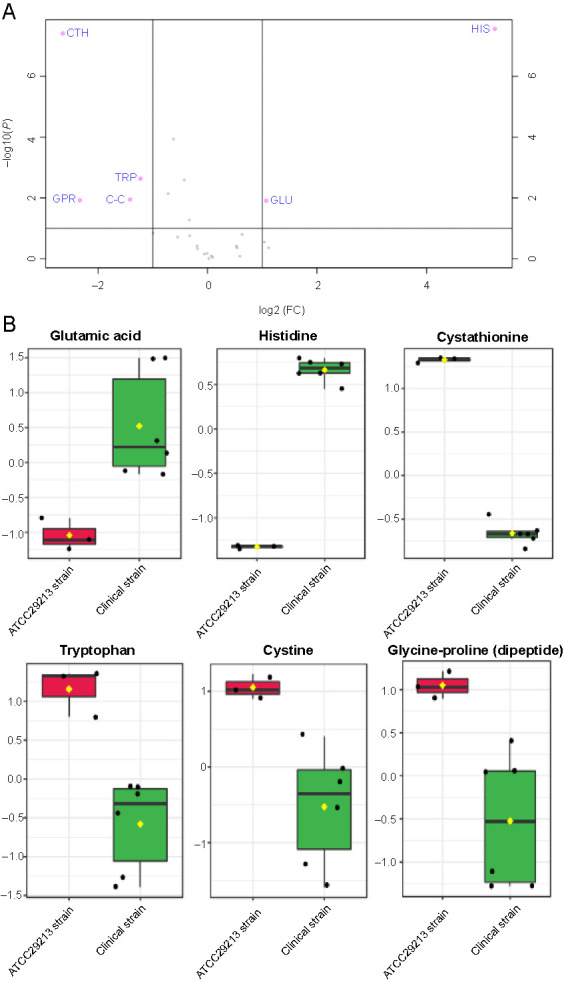
(A) Volcano plot showing amino acid distribution and significance. Light gray dots represent amino acids identified with no significant difference, and pink dots represent amino acids which appeared in different concentrations; (B) Box and whisker plots of the clinical and ATCC 29213 strains demonstrating mean change in intensity values of the altered amino acids between strains. The notch of the plot indicates the median value, the yellow diamond shows the mean value of the feature in the group, and the black dots indicate replicates for each strain. FC: Fold change; GLU: Glutamic acid; HIS: Histidine; CTH: Cystathionine; TRP: Tryptophan; C-C: Cystine; GPR: Glycine-proline (dipeptide).

### Stationary phase analysis

The amino acid profiles of both strains cultured to the stationary phase were also compared to investigate where the metabolic homeostasis would be altered under conditions of diminished nutrient supply, toxic waste accumulation, and pH change in the medium, and the comparison was done with equivalent sets. The analyses of the cytoplasmic amino acids of both strains, grown under normal conditions, discovered the incidence of 28 amino acids and amino acid derivatives that could be detected by the GC-FID method. The most abundant cytoplasmic amino acid in both strains was aspartic acid, which accounted for 44% of the total cytoplasmic amino acids in the clinical strain and 59% in the ATCC 29213 strain. Lysine was the next most abundant amino acid in the cytoplasm, accounting for 16% of the total cytoplasmic amino acids in both strains. Glutamic acid was the third most abundant amino acid in both strains, but it was significantly higher in the clinical strain than in the ATCC 29213 strain. Other significant differences were observed in the cytoplasmic profiles of amino acids, as shown in the volcano plot (Figure 5A). For example, histidine was substantially higher in concentration in the clinical strain than in the ATCC 29213 strain, where it was virtually absent, with concurrent lower levels of tryptophan, cystine, cystathionine, and glycine–proline. Box and whisker plots for significantly altered amino acids between strains were generated from the volcano plot, which is a combination of fold change (FC > 2.0) and *P* value (*P* < 0.1) false discovery rate and equal group variance (Figure 5B).

**Figure 6.**
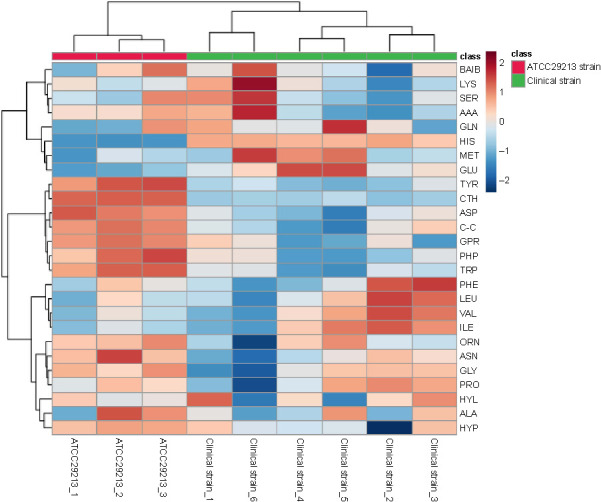
**Heatmap analysis of intracellular amino acids analyzed from the cells cultured to the stationary phase.** Rows represent amino acids and columns represent the replicates for each strain. The different colors of the heatmap depict the relative fold change of each amino acid between the clinical and ATCC 29213 strains. Red and blue colors represent higher or lower levels, respectively. BAIB: β-aminoisobutyric acid; LYS: Lysine; SER: Serine; GLN: Glutamine; HIS: Histidine; MET: Methionine; GLU: Glutamic acid; TYR: Tyrosine; CTH: Cystationine; ASP: Aspartic acid; TPR: Tryptophan; PHE: Phenylalanine; LEU: Leucine; VAL: Valine; ILE: Isoleucine; ORN: Ornithine; ASN: Aspargine; GLY: Glycine; PRO: Proline; HYL: Hydroxylysine; ALA: Alanine; HYP: Hydroxyproline; PHP: Proline-hydroxyproline (dipeptide); C-C: Cystine; GPR: Glycine-proline (dipeptide); AAA: α-aminoadipic acid.

To further compare the amino acid profiles of clinical and ATCC 29213 strains harvested at the stationary phase, a heatmap was generated to visually represent the amino acid abundances in each replicate of both strains (Figure 6). The concentrations of amino acids were ranked by the *t*-test/ANOVA option. The analysis revealed accurate group clustering and the dendrogram structure, using Euclidean distance and Ward linkage, resulting in two main clusters associated with examined strain replicates and amino acids, respectively. The heatmap exhibited distinct patterns of amino acid profiles between the clinical and ATCC 29213 strains. Thus, amino acid patterns may be used to differentiate between *S. aureus* strains.

**Figure 7.**
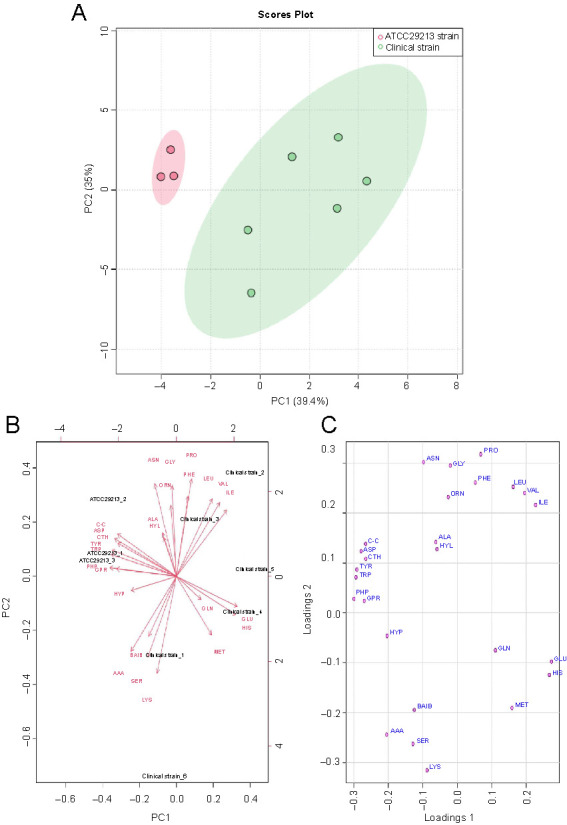
(A) PCA scores (PC1 vs PC2) plotted from cytoplasmic amino acid data analyzed from the stationary phase for clinical (green oval) and ATCC 29213 (pink oval) strains of *S. aureus*. The *S. aureus* strain cultures were grown to the stationary phase prior amino acids analysis by GC-FID; (B and C) Loadings plot and biplot derived from PCA model. PCA: Principal component analysis; GC-FID: Gas chromatography flame ionization; BAIB: β-aminoisobutyric acid; LYS: Lysine; SER: Serine; GLN: Glutamine; HIS: Histidine; MET: Methionine; GLU: Glutamic acid; TYR: Tyrosine; CTH: Cystationine; ASP: Aspartic acid; TRP: Tryptophan; PHE: Phenylalanine; LEU: Leucine; VAL: Valine; ILE: Isoleucine; ORN: Ornithine; ASN: Aspargine; GLY: Glycine; PRO: Proline; HYL: Hydroxylysine; ALA: Alanine; HYP: Hydroxyproline; PHP: Proline-hydroxyproline (dipeptide); C-C: Cystine; GPR: Glycine-proline (dipeptide); AAA: α-aminoadipic acid.

**Figure 8.**
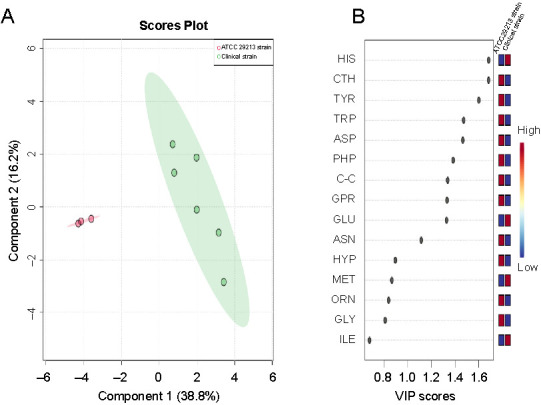
(A) PLS-DA score plot for comparison of the amino acid patterns of clinical strain (green) and ATCC29213 (red) strain after being grown to stationary phase; (B) VIP plot showing the key amino acid features identified by PLS-DA for both strains harvested at the stationary phase. Colored boxes specify concentration of matching amino acids for replicates. PLS-DA: Partial least squares-discriminant analysis; VIP: Variable importance projection; HIS: Histidine; CTH: Cystathionine; TYR: Tyrosine; TRP: Tryptophan; ASP: Aspartic acid; GLU: Glutamic acid; ASN: Asparagine; HYP: Hydroxyproline; MET: Methionine; ORN: Ornithine; GLY: Glycine; ILE: Isoleucine; PHP: Proline-hydroxyproline (dipeptide); C-C: Cystine; GPR: Glycine-proline (dipeptide).

In addition, the cytoplasmic amino acid datasets obtained from both strains were subjected to PCA to assess the variance in rates and differences resulting in the amino acid profiles that characterize the clinical and ATCC 29213 strains. The PCA score plot revealed a clear distinction between the analyzed data, and PC1 and PC2 were explained as 39.4% and 35%, respectively (Figure 7A). The loadings plot revealed the amino acids that were responsible for clustering the strains into two groups in the PCA model (Figure 7B). The biplot is a combination of PCA and loadings plot, showing the position of each replicate of both strains and the position of amino acids which contribute to the distinction between the clinical and ATCC 29213 strains (Figure 7C). In the assessment of the substantial separation accomplished by PCA, PLS-DA was consequently implemented to exploit the differences and identify other important amino acids to those recognized by PCA. In the PLS-DA score plot, the resolving between different *S. aureus* strains was more noticeable (Figure 8A). According to PLS-DA, the variations between the two strains occurred mostly along component 1, measured as 38.8%. The VIP scores generated from PLS-DA demonstrated the 15 most important amino acids in discriminating between the amino acid profiles of the two strains in the PLS-DA score plots (Figure 8B). These include histidine, cystathionine, tyrosine, tryptophan, aspartic acid, phenylalanine, cystine, glycine–proline, and glutamic acid. The colored boxes show the concentration of the equivalent amino acids in the clinical and ATCC 29213 strains, with the red and blue colors indicating high and low concentrations, respectively. This analysis exhibited that the samples were grouped dependent on the *S. aureus* strain, demonstrating great reproducibility within replicates with distinct amino acid patterns amongst strains.

## Discussion

The outcomes from the present study provided clear evidence that the cytoplasmic amino acid profiles observed in the clinical strain were different from those observed in the ATCC 29213 strain at the mid-exponential and stationary growth phases under optimal growth conditions. The altered cytoplasmic profiles of amino acids were strain-specific for each of the growth phases. These results supported the hypothesis that different strains of *S. aureus* would have different amino acid profiles, suggesting a different adaptation mechanism in the transition between the two growth phases, including depleted nutrients and accumulated waste products. These observations suggest that the altered amino acid profiles of *S. aureus* strain could be the outcome of complex evolutionary processes that are beyond our present understanding. Therefore, the current study provided a scenario that revealed how different strains of *S. aureus* would be similar and divergent in terms of amino acid levels at the mid-exponential phase and stationary phase. The outcomes of the current study were interpreted to indicate that changes in metabolic homeostasis were required to adapt to the different growth conditions with different responses elicited by the two strains, suggesting that each strain had its own characteristic mechanisms of adaptation to achieve ideal homeostasis for survival.

Amino acids are crucial elements for nitrogen and carbon metabolism in bacteria and represent important precursors for the synthesis of cell wall peptidoglycan, phospholipids, nucleotides, and proteins [24]. Various bacterial strains may have different abilities to catabolize amino acids depending on their genetic materials and mechanisms to acclimatize to alterations in the environmental conditions present in different growth phases. This results in significant variations in their phenotypes, although they are classified to the same species. Bacterial strains that were resistant to different antibiotics revealed distinct preferences for the consumption of certain amino acids [25]. On this basis, it has been suggested that metabolic fingerprinting could be a new method to determine the classification of bacterial strains and biochemical characteristics [25]. A recent study showed that changes in environmental conditions led to distinct profiles of amino acid uptake and release in the clinical strain following growth to the mid-exponential and stationary phases [6]. The results indicated that the cytoplasmic composition of amino acids in the clinical strain was significantly different from that of the ATCC 29213 strain grown under optimal conditions and harvested at the mid-exponential growth phase. Both strains shared common features with glutamic acid, aspartic acid, proline, and alanine as major components, but the concentration profiles showed significant differences for seven of the amino acids, even though the total cytoplasmic levels of amino acids did not alter significantly. Different amino acid uptake profiles were observed among different strains of *S. aureus* [25]. Various metabolic responses were also observed in MRSA and MSSA following exposure to antibiotics [8]. This phenomenon was deciphered as a possible different stress response mechanism in MRSA and MSSA metabolism [8]. Both strains used in this study had previously shown an independent response to changes in environmental conditions, including variations in pH, salt, and temperature [1, 2]. A previous study found a novel variant of the staphylokinase gene in the ATCC 29213 strain [26]. It has been demonstrated that variations of the core genome within *S. aureus* strains can have major effects on the virulence, replications and adaptations, as well as metabolism of these strains [27–29]. Therefore, a more in-depth investigation of strain-specific metabolic alterations could offer additional insights into the survival and pathogenicity of *S. aureus*. The clinical strain had more glycine compared to the ATCC 29213 strain in the cells collected at the mid-exponential phase. This might be associated with the development of pentaglycine cross-bridge, required for peptidoglycan cell wall biosynthesis; the absence of a pentaglycine cross-bridge would hinder the survival of the clinical strain. Previous studies showed that the clinical strain developed a thicker cell wall in response to altered environmental conditions [30, 31]. These observations indicate that this is a response mechanism to combat changes in the external environment. Prior publications have shown that the clinical strain underwent significant alterations in its cell wall and colony size, forming small colony variants, and was resistant to commonly used antibiotics [30, 31]. It also responded to wound site conditions by inducing substantial alterations in metabolites and proteins [32]. These features are similar to those found in MRSA strains [8]. Thus, this study suggested that each *S. aureus* strain would respond differently to changes in growth conditions. In fact many recent studies have demonstrated significant differences between *S. aureus* strains at the genome and proteome levels [33, 34]. Consequently, this study would lineup with previous publications to strengthen our knowledge in bacterial biology, which would ultimately lead to clinical implications.

The amino acid profiles of both strains were also investigated at the stationary phase of growth, where the metabolic homeostasis is adjusted under conditions of reduced nutrient supply, accumulation of toxic waste, and pH change in the medium. This investigation has shown that the shift to the extended stage of growth resulted in different amino acid patterns of the clinical strain in comparison to the ATCC 29213 strain. This outcome indicated that changes in metabolic homeostasis were required to adapt to changes present in the stationary phase, and the different adjustment in amino acid profiles provoked by the two strains showed that each strain had its own specific mechanisms for acclimation to achieve an optimal metabolic homeostasis for survival. These changes in amino acids were certainly due to the change in the environmental conditions, including nutrient limitation, toxic waste accumulation, and pH change. The amino acids also represent activity snapshots of various biochemical pathways utilized in their synthesis. The precise roles of the altered states of biochemical homeostasis were not obvious at this stage, but the relative abundances within the profile were consistent between replicates of each strain at the growth stages and reproducible across repeated experiments. Other, less abundant amino acids, such as tryptophan, cystine, and cystathionine, were also clearly discriminant between the clinical and ATCC 29213 strains. These results suggested that the governing amino acid profile in the cytoplasm was not simply a reflection of leftover resources from protein production, but contributed to ongoing metabolism and nitrogen balance.

Aspartic acid, lysine, and glutamic acid were the most abundant cytoplasmic components in both strains at the stationary phase, with glutamic acid being higher in the clinical strain. Histidine was only present in the clinical strain and was almost absent in the ATCC 29213 strain. Similar patterns of lysine and histidine were noted in the *S. aureus* COL strain during growth at the stationary phase in response to nutrient limitation [35]. Thus, the results of the current study suggest that histidine could be used for classification between the clinical strain and the ATCC 29213 strain. Differences in metabolites were also observed in *Pseudomonas aeruginosa* strains. The differences in metabolites in bacterial strains could be a result of the specificity of the strain to grow, survive, and ultimately cause infections [36, 37]. It has been shown that metabolites could be used as markers for the determination of effective and ineffective antibiotics against *S. aureus* infections [38]. The different strategies for adaptation and survival applied by the two strains during exposure to the accumulation of toxins and acidity at the stationary phase suggested that each strain may also have a unique approach to invasion, infection, and evasion of the host defense mechanisms. These findings support the hypothesis that the bacterium possesses a considerably large redundancy in the metabolic pathways that ensure survival in response to external threats and that there are subtle differences in the redundancy mix of pathways between strains of *S. aureus*. Thus, when a snapshot of amino acids is taken at one point in time, significantly different phenotypic profiles are seen between the strains. Understanding this redundancy will lead to an understanding of how these organisms have survived for billions of years and will also lead to novel methods of controlling the bacteria in the clinical setting.

The specific adjustment of amino acid concentrations may reflect a range of demands for protein synthesis where altered sets of proteins would be required for each strain to optimize the conditions induced by different growth phases. Some amino acids could also be used for the synthesis of other compounds, such as fatty acids and nucleotides. It could also be suggested that the different responses of strains to different growth phases are essential for the adaptation of strains to environmental changes during various growth stages [39]. A prior study showed that different strains of *S. aureus* utilize and secrete different quantities of amino acids [16]. A recent investigation indicated that the clinical strain of *S. aureus* consumed different levels of amino acids in response to various environmental conditions and throughout various growth stages [6]. The different utilization and secretion of amino acids could suggest that alternative mechanisms were required by each strain to obtain an optimal metabolic homeostasis. It was proposed that these alterations in the consumption of amino acids, necessary to support adaptation to the changing environment, occur during different growth stages. The cytoplasmic changes are part of a whole-cell adaptation mechanism and include changes in cell size, membrane composition, cytoplasmic composition, and proteome. Previous investigations of the clinical and ATCC 29213 strains have indicated that subtle variations in environmental conditions elicit responses that can be attributed to the cytoplasmic amino acids specific to the prevailing growth conditions [2]. On this basis, a premise was anticipated whereby the bacterium persistently detects and regulates the environmental conditions prevailing in the different growth phases to yield the most effective phenotypes for ideal growth in the various growth stages.

## Conclusion

This investigation provided convincing evidence that *S. aureus* strains used in this study had very different cytoplasmic amino acid compositions at the mid-exponential and stationary phases, as assessed by multivariate analyses, including PCA and PLS-DA. The results of this study illustrated that various growth phases led to substantial changes in the amino acid profiles of the clinical and ATCC 29213 strains of *S. aureus*. The alterations in amino acid levels were reproducible, strain-specific, and growth phase-specific. The clinical and ATCC 29213 strains displayed some similarities in amino acid composition, but each strain had characteristic amino acid profiles and different strategies for achieving metabolic homeostasis at the mid-exponential and stationary phases.
